# The non-volatile electrostatic doping effect in MoTe_2_ field-effect transistors controlled by hexagonal boron nitride and a metal gate

**DOI:** 10.1038/s41598-022-16298-w

**Published:** 2022-07-15

**Authors:** Muhammad Asghar Khan, Muhammad Farooq Khan, Shania Rehman, Harshada Patil, Ghulam Dastgeer, Byung Min Ko, Jonghwa Eom

**Affiliations:** 1 Department of Physics and Astronomy, and Graphene Research Institute-Texas Photonics Center International Research Center (GRI–TPC IRC), Sejong University, Seoul, 05006 Korea; 2 Department of Electrical Engineering, Sejong University, Seoul, 05006 Korea; 3 Department of Convergence Engineering for Intelligent Drone, Sejong University, Seoul, 05006 Korea

**Keywords:** Materials science, Nanoscience and technology

## Abstract

The electrical and optical properties of transition metal dichalcogenides (TMDs) can be effectively modulated by tuning their Fermi levels. To develop a carrier-selectable optoelectronic device, we investigated intrinsically p-type MoTe_2_, which can be changed to n-type by charging a hexagonal boron nitride (h-BN) substrate through the application of a writing voltage using a metal gate under deep ultraviolet light. The n-type part of MoTe_2_ can be obtained locally using the metal gate pattern, whereas the other parts remain p-type. Furthermore, we can control the transition rate to n-type by applying a different writing voltage (i.e., − 2 to − 10 V), where the n-type characteristics become saturated beyond a certain writing voltage. Thus, MoTe_2_ was electrostatically doped by a charged h-BN substrate, and it was found that a thicker h-BN substrate was more efficiently photocharged than a thinner one. We also fabricated a p–n diode using a 0.8 nm-thick MoTe_2_ flake on a 167 nm-thick h-BN substrate, which showed a high rectification ratio of ~ 10^−4^. Our observations pave the way for expanding the application of TMD-based FETs to diode rectification devices, along with optoelectronic applications.

## Introduction

Graphene, which is one of the most intriguing two-dimensional (2D) materials for electronic applications due to its high electron mobility, flexibility, thermal conductivity, large surface area, and impermeability to gases, has been studied extensively over last two decades^1–8^. Despite its many merits for use in electronic materials, the application of graphene for switching devices is restricted due to its gapless nature in the pristine state^9^. However, transition metal dichalcogenides (TMDs), which are 2D semiconductor materials, exhibit a wide range of doping and band structure dynamics, allowing them to be used in a broad range of optoelectronics and nanoelectronics^10–13^. TMDs are composed of atomic layers bound together by van der Waals forces^14^ and have good electronic transport channels with minimal scattering centers because they do not possess any interlayer covalent bonds^15,16^. Therefore, the bandgap and atomically thin layered structure of 2D TMDs render them a viable material for the active channel of field-effect transistor applications, such as ultra-fast photodetectors^17^, electro- and photo-catalysis, supercapacitors^18^, biosensors, energy storage devices, and memory devices, among others ^19–23^.

In the context of TMDs for use in electronic material applications, MoTe_2_ has gained significant interest owing to its fascinating semiconducting, metallic, and superconducting characteristics^24–27^. The direct bandgap of MoTe_2_ varies between 0.88 and 1.1 eV depending on the lattice configuration and number of layers^28–30^. In addition, since the bandgap of MoTe_2_ is significantly smaller than those of MoS_2_^31,32^ and WSe_2_^33,34^, MoTe_2_ is a good candidate for optoelectronic devices that provide a response covering the near-infrared wavelength region^35^. Moreover, in comparison to sulfur-terminated TMDs, Fermi-level pinning at the MoTe_2_-metal interface is significantly weaker^36^. Despite the narrow energy bandgap of this material, numerous methods have been reported for band modulation and control of the charge carrier polarity^37,38^. On the other hand, hexagonal boron nitride (h-BN), an insulating 2D material, has recently attracted growing interest because of its mechanical robustness, its exceptional thermal conductivity due to its strong BN covalent bonds, and its donor/acceptor-like defect states that control the doping mechanism^39,40^.

It is therefore important to develop an efficient doping method for 2D TMDs to promote their application in semiconducting electronic applications. In this context, the doping of MoTe_2_ can be categorized into two types. The first method employs local electrostatic gating, which has been used successfully to create a p–n junction by polarizing a local area where the charge carrier type is opposite to that in other parts of the MoTe_2_ flake^41,42^. Although this technique is extremely adaptable, it is particularly volatile when gate voltage is turned off. The second method consists of atomic doping and surface modification using physical and chemical processes^43,44^. These processes permanently transform the material; however, but p-type and n-type doping are difficult to combine in the local areas of a single device. There is another way to to manupolate the carrier’s type in MoTe_2_. This method involves metal contacts engineering, which makes use of low and high work function metal electrodes^45,46^. For example, platinum, which is a high work function metal, has been used for as a source and drain contact and ambipolar MoTe_2_ was converted into a unipolar p-type field-effect transistor (FET)^47^. However, the unipolar n-type transport of MoTe_2_ is exceedingly difficult to accomplish owing to Fermi-level pinning and a limited variety of low work function metals. Thus, to modulate the carrier type and concentration in MoTe_2_, the development of a stable, nonvolatile, and controlled technique is necessary to adjust the properties of MoTe_2_ from the broad perspective of electronic devices.

Here, we present a promising strategy to address the aforementioned difficulties. More specifically, we employ a localized metal gate on a specific region of MoTe_2_, wherein h-BN is used as a dielectric material in the metal gate, and its thickness plays a vital role in the electrostatic doping of MoTe_2_. One region of the MoTe_2_ is placed on an h-BN substrate with a localized metal gate underneath, while the other region is placed on h-BN without a gate to allow control of the gate effect on a specific region of the MoTe_2_. Subsequently, illumination with deep ultraviolet (DUV) light is carried out to induce charge transfer to the defect states of h-BN with the localized metal gate underneath. Then, h-BN with charged defect states functions as a gate electrode to cause electrostatic doping of the localized MoTe_2_ region. We also investigate the characteristics of p–n diodes consisting of p-MoTe_2_ and n-MoTe_2_, which are fabricated using h-BN and a metal gate.

## Results and discussion

### Photo-induced doping effect of h-BN/MoTe_2_ FET

**Figure 1:**
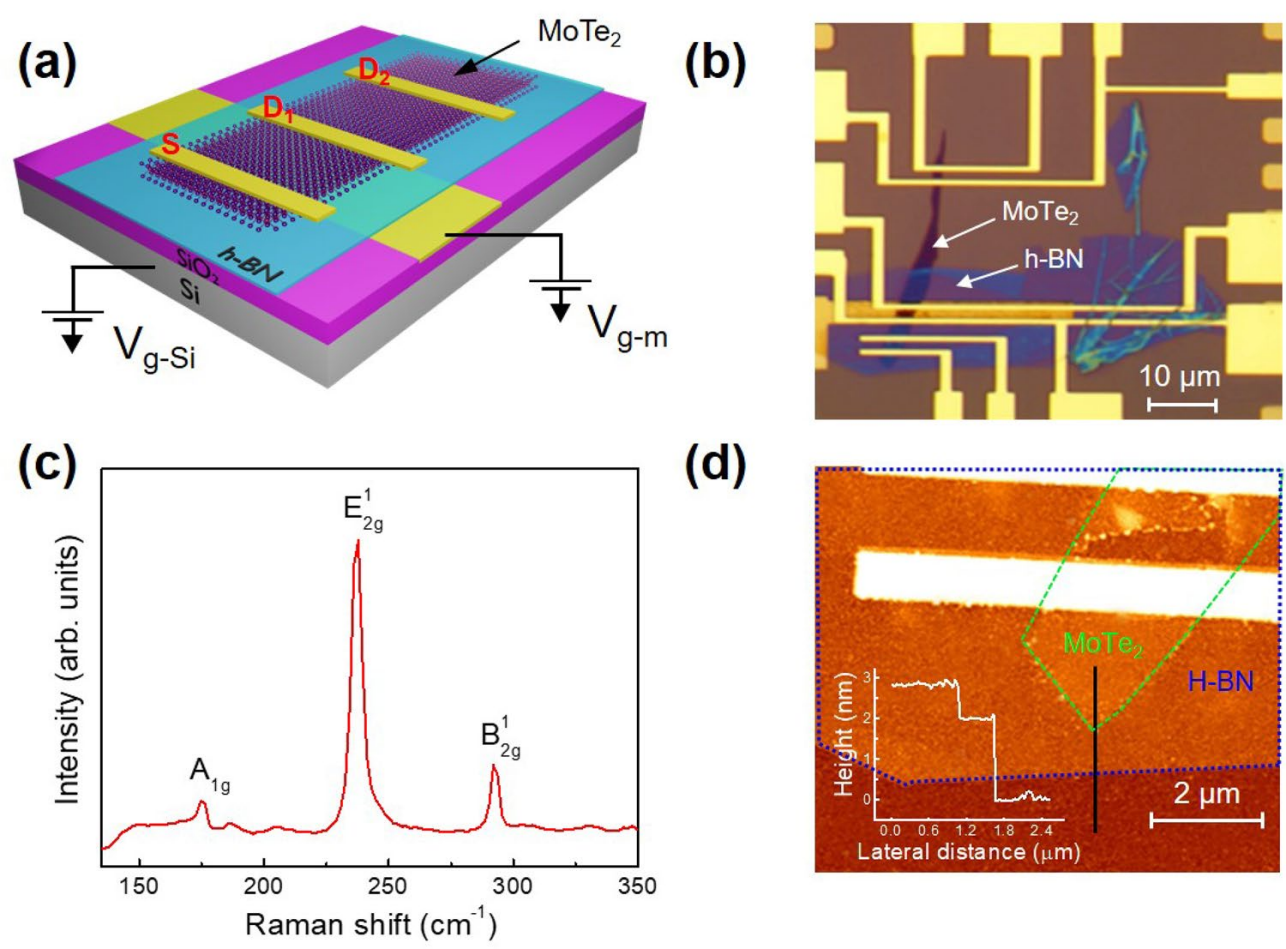
(**a**) Schematic diagram of an h-BN/MoTe_2_ FET. (**b**) Optical image of an h-BN/MoTe_2_ FET. (**c**) Raman spectrum of MoTe_2_. (**d**) AFM image and height profile of an h-BN/MoTe_2_ FET.

h-BN and MoTe_2_ nanoflakes were fabricated using adhesive tape and a conventional mechanical exfoliation process, and the dry transfer technique was used to prepare stacks of the h-BN/MoTe_2_ heterostructures. Figure 1a,b show a schematic diagram and an optical microscope image of the h-BN/MoTe_2_ heterostructure-based FET, respectively. We also examined the 2D flakes using Raman spectroscopy, which is a non-destructive and precise technique for determining the strain effect, thermal conductivity, band structure, and adsorption of chemicals on material surfaces^48–50^. To prevent the heating effect, the Raman spectra were recorded at room temperature using a laser with a wavelength of 514 nm and a low laser power of 1.0 mW. Figure 1c shows the Raman spectra of MoTe_2_ and three peaks assigned to A_1g_ (174.63/cm), E^1^_2g_ (237.87/cm), and B^1^_2g_ (291.97/cm). The Raman spectra of h-BN are provided in supplementary information Fig. S1, where we observed a dominant E_2g_ peak (1364.47/cm). Figure 1d shows the topographical atomic force microscopy (AFM) image and height profile of the h-BN/MoTe_2_ heterostructure, indicating that the thicknesses of the h-BN and MoTe_2_ components were 2 and 0.8 nm, respectively.

**Figure 2:**
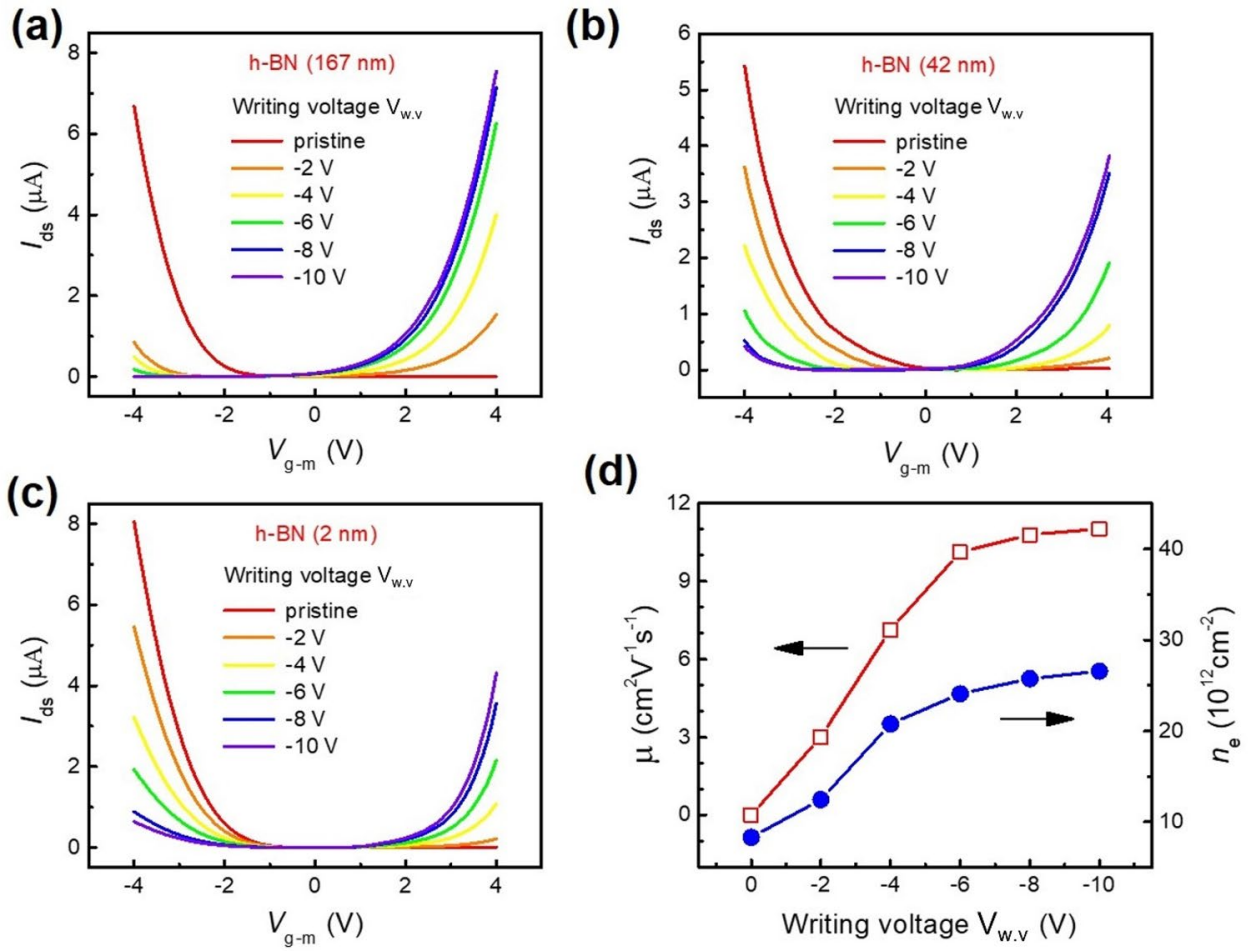
(**a**) Transfer characteristics of MoTe_2_ (0.8 nm) FET on a 167 nm-thick h-BN substrate before and after photo-induced doping under DUV illumination (5 min) with writing voltages ranging from − 2 to − 10 V. (**b**) Transfer characteristics of the MoTe_2_ (2.4 nm) FET on a 42 nm-thick h-BN substrate. (**c**) Transfer characteristics of the thin MoTe_2_ (1.6 nm) FET on a 2 nm-thick h-BN substrate. (**d**) Electron mobility and carrier concentration of the MoTe_2_ (0.8 nm) FET on a 167 nm-thick h-BN substrate after photo-induced doping with different metal gate voltages.

The charge carrier type of a TMD plays an important role in the interface resistance between the contact metal and semiconductor. Pristine MoTe_2_ can be either ambipolar or unipolar, being n-type or p-type, depending on its natural doping state^36,51–56^. We found that our thin MoTe_2_ flakes were p-type in the pristine state. Thus, we initially fabricated a thin layer of MoTe_2_ (0.8 nm) on a thick h-BN layer (167 nm). A Si/SiO_2_ substrate was employed, where Si was degenerately doped for use as the back gate. The AFM images and height profiles of both h-BN and MoTe_2_ are shown in supplementary information Fig. S2. Pristine MoTe_2_ (0.8 nm) was found to exhibit p-type behavior, as shown in the transfer curves (*I*_ds − _*V*_g−m_) and (*I*_ds _− *V*_g−Si_) given in Fig. 2a and supplementary information Fig. S3a, respectively. During the transfer curve measurements, which were performed in a vacuum, the drain-source voltage (*V*_ds_) was fixed at 0.5 V. In addition, we have investigated the output characteristics of pristine thin p-type MoTe_2_ and found that I–V curves are nonlinear as shown in Fig. S3b, which indicates the existence of a Schottky barrier between thin MoTe_2_ and metal contact (Cr/Au). Subsequently, the photo-induced doping effect was investigated when h-BN/MoTe_2_ was illuminated by DUV for various time intervals with the application of a writing voltage (*V*_w.v_) ranging from − 2 to − 10 V, as shown in Fig. 2a. The writing voltages are applied through a localized metal gate (Cr/Au, 3/13 nm) to fill or deplete electrons in the defect sites of the h-BN layer with the help of a DUV in a vacuum. To achieve this photo-induced doping effect, the use of both a DUV and a writing voltage are essential^57^. Figure 2a shows a pristine MoTe_2_ FET on h-BN that was initially p-type, but that had been converted into n-type by DUV illumination and the application of a writing voltage. Initially, the application of − 2 V writing voltage under DUV light illumination resulted in a change in the polarity of the pristine MoTe_2_ from p-type to n-type, as shown in Fig. 2a. Upon further increasing the writing voltage, the MoTe_2_ region above the localized metal gate became completely n-type at a − 10 V writing voltage^58–60^. In addition, higher writing voltages resulted in more positive charges on the h-BN flake, which eventually provided an additional positive gate voltage. This photo-induced doping effect of MoTe_2_ can be attributed to a mechanism involving the electron depletion of donor-like defects in the h-BN flakes, which are generated by the negative gate voltage upon DUV optical excitement^61,62^. The depleted electrons enter the conduction band of the h-BN and then transfer to the MoTe_2_, leaving positively ionized defects inside the h-BN layer, which can be observed under an external electric field (*V*_*g−m*_). Consequently, these positively charged donor-like defects in the h-BN resulted in the electrostatic doping effect of MoTe_2_.

### Effect of the h-BN thickness

To investigate whether donor-like defects exist at the h-BN/MoTe_2_ interface or inside the h-BN itself, we measured the photo-induced doping characteristics of MoTe_2_ films with various h-BN thicknesses. If the photo-induced doping rate is proportional to the h-BN thickness, then it can be assumed that the defects exist inside the h-BN body; however, if the photo-induced doping effect originates from defects at the interface, it must be independent of the h-BN thickness. Thus, we fabricated thin (0.8–2.4 nm) MoTe_2_ FETs with different h-BN thicknesses to reveal the role of the h-BN thickness in the photo-induced doping effect. The transfer characteristics of the MoTe_2_ (2.4 nm)/h-BN(42 nm) heterostructure were measured with a drain-source voltage of 0.5 V and a metal gate voltage from − 4 to + 4 V as shown in Fig. 2b. AFM confirmed the thicknesses of the MoTe_2_ and h-BN layers, as shown in supplementary information Fig. S4. As the writing voltage was increased from − 2 to − 10 V, the polarity of MoTe_2_ changed from p-type towards n-type, but it did not convert completely to n-type, remaining ambipolar. Similarly, the transfer characteristics of another MoTe_2_ (1.6 nm thickness) FET on a thin (2 nm) h-BN layer were evaluated and are shown in Fig. 2c. In this case, we also observed that the p-type pristine MoTe_2_ did not completely change its polarity to n-type and again remained ambipolar. The photo-induced doping effect rates in Fig. 2b,c are in contrast to those in Fig. 2a, where the underlying h-BN flakes are particularly thick. Our findings therefore imply that photo-induced doping in h-BN/MoTe_2_ heterostructures is attributed to the optical stimulation of electronic states within the h-BN layer, and the thickness of this h-BN layer plays an important role in determining the extent of photo-induced doping. It is also possible that donor-like defect states may exist at different depths inside the h-BN flakes; Fig. S5 in supplementary information shows a schematic representation of the remaining positive defects in thin and thick h-BN layers after DUV illumination with the application of a writing voltage. Since DUV is illuminated from the top side of the h-BN flake, the positive defects are found more in the upper part of the h-BN flake. To compare the photo-induced doping effect at different writing voltages, we estimated the carrier density of the MoTe_2_ FET. The charge-carrier concentration (*n*_e_) can be calculated as follows^63^:

$$n_{e} = \frac{{C_{g} \left( {V_{g - m} - V_{th} } \right)}}{e},$$

where *V*_th_ is the electron transport threshold voltage, *V*_g−m_ is the metal gate voltage, and *e* is the charge of an electron (1.602 × 10^−19^ C). The capacitance value (*C*_g_) of h-BN per unit area can be calculated as *C*_g_ = ε_0_ ε_r_/*d*, where *d* is the thickness of the h-BN layer, ε_0_ is the vacuum permittivity, and ε_r_ is the dielectric constant of h-BN. Figure S6a in supplementary information shows the gate capacitance vs frequency graphs for different thicknesses of h-BN, which demonstrates that the capacitance decreases with an increasing h-BN layer thickness as shown in Fig. S6b. Figure 2d shows the electron carrier concentration *n*_e_ after photo-induced doping under the application of a writing voltage (*V*_w.v_) in combination with DUV for a MoTe_2_ (0.8 nm) FET on a thick (167 nm) h-BN layer. The carrier concentration (n_e_) was estimated at *V*_g−m_ =  + 4 V after photo-induced doping. Similarly, we estimated n_e_ at *V*_g−m_ = 0 V as shown in Fig. S6c, which shows a similar behaviour but the number of charge carriers is less as compared to n_e_ at *V*_g−m_ = + 4 V. In addition, we calculated the field-effect mobility of the MoTe_2_ FET using the following equation.

$${\upmu } = \frac{L}{W}\left( {\frac{{dI_{ds} }}{{dV_{g - m} }}} \right)\frac{1}{{C_{g} V_{ds} }}$$

where *W* is the channel width, *L* is the channel length, and $\frac{{dI_{ds} }}{{dV_{g - m} }}$ represents the slope of the linear part of the transfer characteristics of the MoTe_2_ FET at an applied *V*_ds_ of 0.5 V. Figure 2d shows the mobility of the MoTe_2_ (0.8 nm) FET on a thick (167 nm) h-BN layer after the application of a writing voltage *V*_w.v_ in combination with DUV. Furthermore, the photo-induced doping effect was found to be stable for several days. The MoTe_2_ FET demonstrated a stable n-type doping effect as shown in supplementary information Fig. S7a.

### Effect of the MoTe_2_ thickness

**Figure 3:**
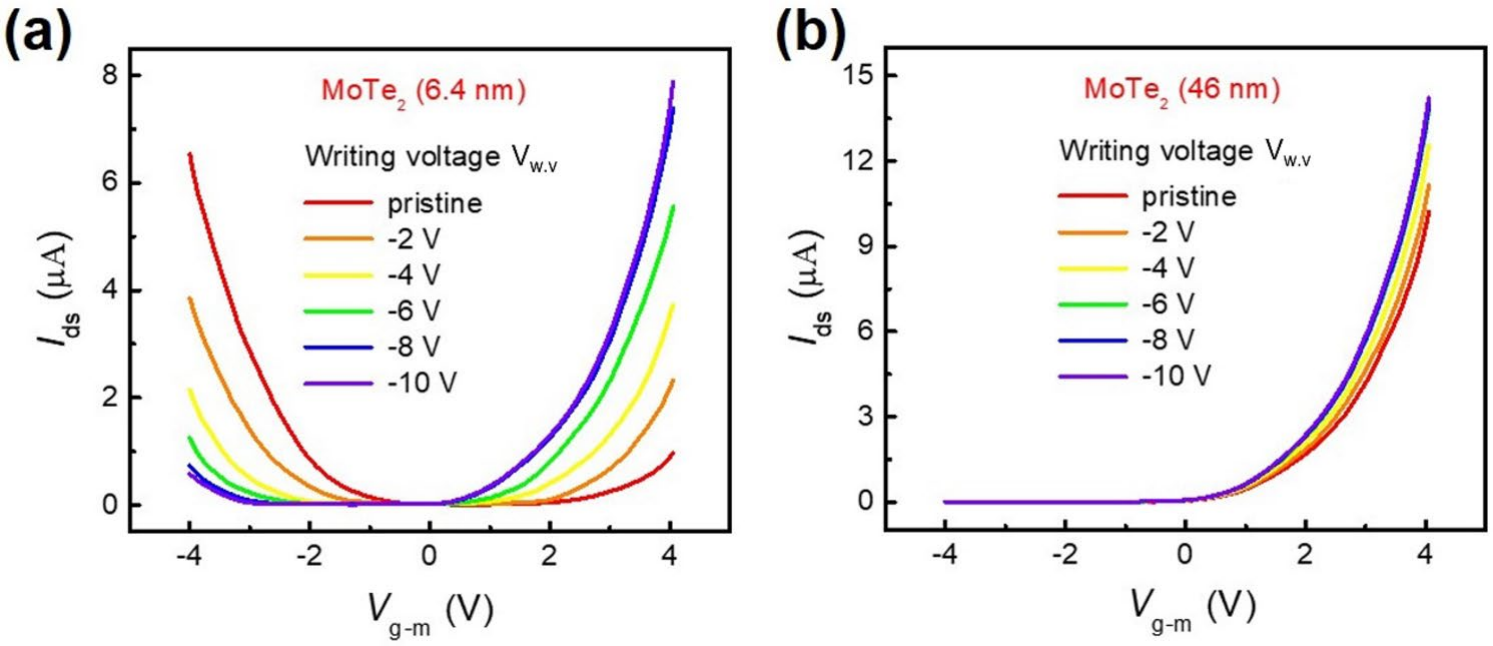
(**a**) Transfer characteristics of the MoTe_2_ (6.4 nm) FET on a 160 nm-thick h-BN substrate. (**b**) Transfer characteristics of the MoTe_2_ (46 nm) FET on a 165 nm-thick h-BN substrate.

We also investigated the dependence of the MoTe_2_ flake on the photo-induced doping effect. For this purpose, two different thicknesses of MoTe_2_ flakes were placed on an h-BN layer, and the transfer curves were measured after photo-induced doping with various writing voltages. Figure 3a shows the transfer curves of the MoTe_2_ (6.4 nm) FET on h-BN (160 nm), which exhibits ambipolar behavior in the pristine state. Further, we have examined the output characteristics of pristine thick n-type MoTe_2_ and found that I–V curves are nonlinear as shown in Fig. S8. However, the writing voltage was increased from − 2 to − 10 V, the n-type characteristics of the MoTe_2_ FET were enhanced after photo-induced doping. For comparison, we examined the photo-induced doping effect in a thicker MoTe_2_ (46 nm) FET on h-BN (165 nm), as shown in Fig. 3b. The transfer curve indicated the n-type characteristics of the pristine MoTe_2_ FET, and it was observed that the photo-induced doping treatment enhanced the n-type properties. More specifically, the pristine MoTe_2_ flake exhibited p-type characteristics when its thickness was < 2.4 nm, as shown in Fig. 2a–c, whereas the thick (46 nm) MoTe_2_ flake exhibited n-type characteristics in the pristine state. These results indicate that a p–n junction can be formed in thin MoTe_2_ flakes using a combination of photo-induced doping treatment and a local metal gate.

### MoTe_2_ p–n diodes with different h-BN thicknesses

**Figure 4:**
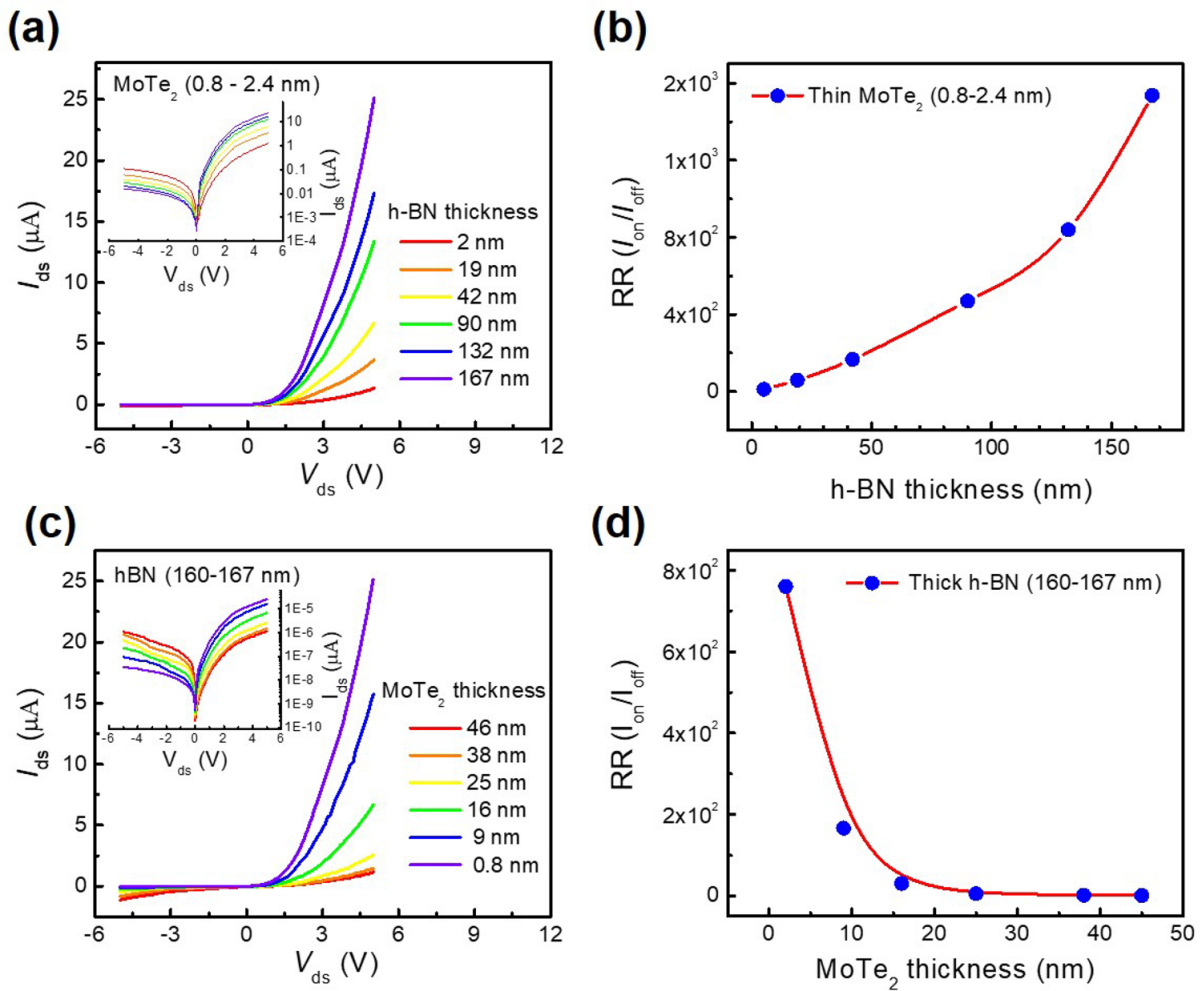
(**a**) Output characteristics of the MoTe_2_ p–n diodes on h-BN substrates of different thicknesses, where the thicknesses of the MoTe_2_ flakes ranged from 0.8 to 2.4 nm. (**b**) Rectification ratio of the MoTe_2_ p–n diodes on h-BN substrates of different thicknesses, where the thicknesses of the MoTe_2_ flakes ranged from 0.8 to 2.4 nm. (**c**) Output characteristics of the MoTe_2_ p–n diodes for MoTe_2_ flakes of different thicknesses. (**d**) Rectification ratio of the MoTe_2_ p–n diodes for MoTe_2_ flakes of different thicknesses, where the thicknesses of the h-BN flakes ranged from 160 to 167 nm.

Subsequently, we employed the photo-induced doping technique to prepare n-type regions at local regions of thin MoTe_2_ flakes (0.8–2.4 nm thick) on h-BN mounted on metal gates, while the other regions remained p-type, similar to the pristine state of MoTe_2_. Although the DUV illuminated the entire area of the h-BN layer, only the donor-like defects at the local regions over the metal gate could be charged. Consequently, a p–n diode was obtained in the MoTe_2_ flake between terminals *S* and *D*_2_, as shown in Fig. 1a. In addition, Fig. 4a shows the output characteristics of the MoTe_2_ p–n diodes with different h-BN thicknesses after photo-induced doping; the inset of Fig. 4a shows the log scale *I*_ds _− *V*_ds_ curves, indicating the rectification characteristics. Since the photo-induced doping rate of MoTe_2_ depends on the thickness of the h-BN layer (see Fig. 2), the function of the p–n diode is expected to also be dependent on this thickness. Figure 4b shows the rectification ratio (RR) of the MoTe_2_ p–n diode for different h-BN thicknesses, where the RR is defined by *I*_on_ at *V*_ds_ = + 5 V divided by *I*_off_ at *V*_ds_ = − 5 V. The highest RR value (~ 1.5 × 10^3^) was found for the MoTe_2_ flake mounted on the thickest h-BN layer (167 nm). We also investigated the MoTe_2_ p–n diode characteristics for different thicknesses of MoTe_2_ flakes. Thus, Fig. 4c shows the output characteristics of the MoTe_2_ p–n diodes with various thicknesses of MoTe_2_, and the inset shows the *I*_ds_−*V*_ds_ curves on a logarithmic scale. As expected, diode characteristics were generally not observed in MoTe_2_ flakes with thicknesses > 16 nm due to the fact that the majority of the flakes will be in the n-type state (i.e., that of the pristine state). As shown in Fig. 4d, a higher RR was achieved for thinner MoTe_2_ flakes.

Following our examination of the photo-induced doping effect with a negative writing voltage of the metal gate, which mainly relies on the presence of donor-like defects in the h-BN layer, we moved on to address the possibility of reverse photo-induced doping. For this purpose, a MoTe_2_ (0.8 nm) FET on an h-BN layer (167 nm) was subjected to DUV illumination for 5 min with a positive writing voltage for the metal gate, as shown in supplementary information Fig. S7b. The same system was used in combination with a writing voltage of − 10 V before starting the experiment, and reverse photo-induced doping was investigated with positive writing voltages ranging from + 2 to + 10 V. It was found that the transfer curve changed toward p-type as the writing voltage increased, but it remained more like n-type even with the highest writing voltage of + 10 V. It should also be noted here that the density of acceptor-like defects was lower than that of the donor-like defect states in the h-BN layer.

## Materials and methods

### Fabrication of MoTe_2_ field-effect transistors on h-BN

The natural bulk crystals of h-BN and MoTe_2_ were provided by HQ graphene. Using adhesive tape in a cleanroom environment, the mechanical exfoliation method was used to obtain ultrathin nanoflakes of h-BN and MoTe_2_ from their bulk forms. A photoresist (SPR) and ethyl lactate (EL) were spin-coated onto Si/SiO_2_ (SiO_2_: 300 nm) substrates in the initial stage of the photolithography process. Subsequently, the obtained patterns were exposed to oxygen plasma for 5 min to eliminate the SPR and EL residues. A thermal evaporator was then used to evaporate Cr/Au (3/30 nm) for the large patterns, while the bottom electrode composed of Cr/Au (3/13 nm) was fabricated using conventional e-beam lithography and thermal evaporation techniques. Subsequently, a large h-BN flake was dry-transferred onto the top of the bottom electrode, while the other remainder was present on the Si/SiO_2_ substrate. The MoTe_2_ flake was then transferred onto the h-BN layer using a micromanipulator, as shown in Fig. S9 in supplementary information. At the end of the transfer procedure, the substrate was placed on a hot plate at 90 °C to eliminate vapor from the external surfaces and interfaces. After each transfer process, the samples were cleaned with acetone and methanol, and finally dried under a flow of N_2_ gas. The source/drain electrodes were fabricated using conventional e-beam lithography. Finally, Cr/Au (10/80 nm) metal contacts were deposited using a thermal evaporation technique.

### Photo-induced doping and measurements

For the photo-induced doping treatment, the MoTe_2_ FETs on h-BN were illuminated by DUV light (λ = 220 nm, 11 mW cm^−2^). Optical microscopy and Raman spectroscopy were used to examine the MoTe_2_ flakes, and their thicknesses were measured by AFM. The electrical transport properties were measured in a vacuum using a source meter (Keithley 2400) and a picoammeter (Keithley 6485).

## Conclusion

We herein reported the fabrication of MoTe_2_ field-effect transistors (FETs) on hexagonal boron nitride (h-BN) with a localized metal gate and found that the photo-induced doping treatment was most effective for thinner MoTe_2_ flakes mounted on a thicker h-BN layer. The use of a negative writing voltage under deep-ultraviolet (DUV) illumination induced n-doping of the MoTe_2_ FET, while the use of a positive writing voltage under DUV illumination induced p-doping; this difference was attributed to the donor- and accepter-like defects present in the h-BN. In addition, it was found that the photo-induced doping effect became stronger as the writing voltage was increased. Furthermore, a negative writing voltage resulted in a stronger doping effect than a positive writing voltage, which indicates that donor-like defects are more dominant than acceptor-like defects in the h-BN. These observations clearly demonstrate the success of this selectable local doping technique, which is applicable as a post-fabrication treatment method.

## Supplementary Information

Supplementary Figures.

## Data Availability

The data that support the findings of this study are available upon reasonable request from the corresponding author.
