# Supplementary material for: The non-volatile electrostatic doping effect in MoTe2 field-effect transistors controlled by hexagonal boron nitride and a metal gate
Source: Sci Rep. 2022 Jul 15;12:12085. doi: 10.1038/s41598-022-16298-w (PMC9287407; doi:10.1038/s41598-022-16298-w)
Supplement: Supplementary file 1 — Supplementary Figures. [file 41598_2022_16298_MOESM1_ESM.docx]

**Supplementary Information**

**The non-volatile electrostatic doping effect in MoTe_2_ field-effect transistors controlled by hexagonal boron nitride and a metal gate**

Muhammad Asghar Khan^1#^, Muhammad Farooq Khan^2#^, Shania Rehman^2,3^, Harshada Patil^2,3^, Ghulam Dastgeer^1^, Byung Min Ko^1^ and Jonghwa Eom^1*^

*^1^Department of Physics & Astronomy, and Graphene Research Institute-Texas Photonics Center International Research Center (GRI–TPC IRC), Sejong University, Seoul 05006, Korea*

*^2^Department of Electrical Engineering, Sejong University, Seoul 05006, Korea*

*^3^Department of Convergence Engineering for Intelligent Drone, Sejong University, Seoul 05006, Korea*

^#^These authors contributed equally.

*Corresponding author: eom@sejong.ac.kr

**Figure S1.** Raman spectrum of the 2 nm-thick h-BN.

**
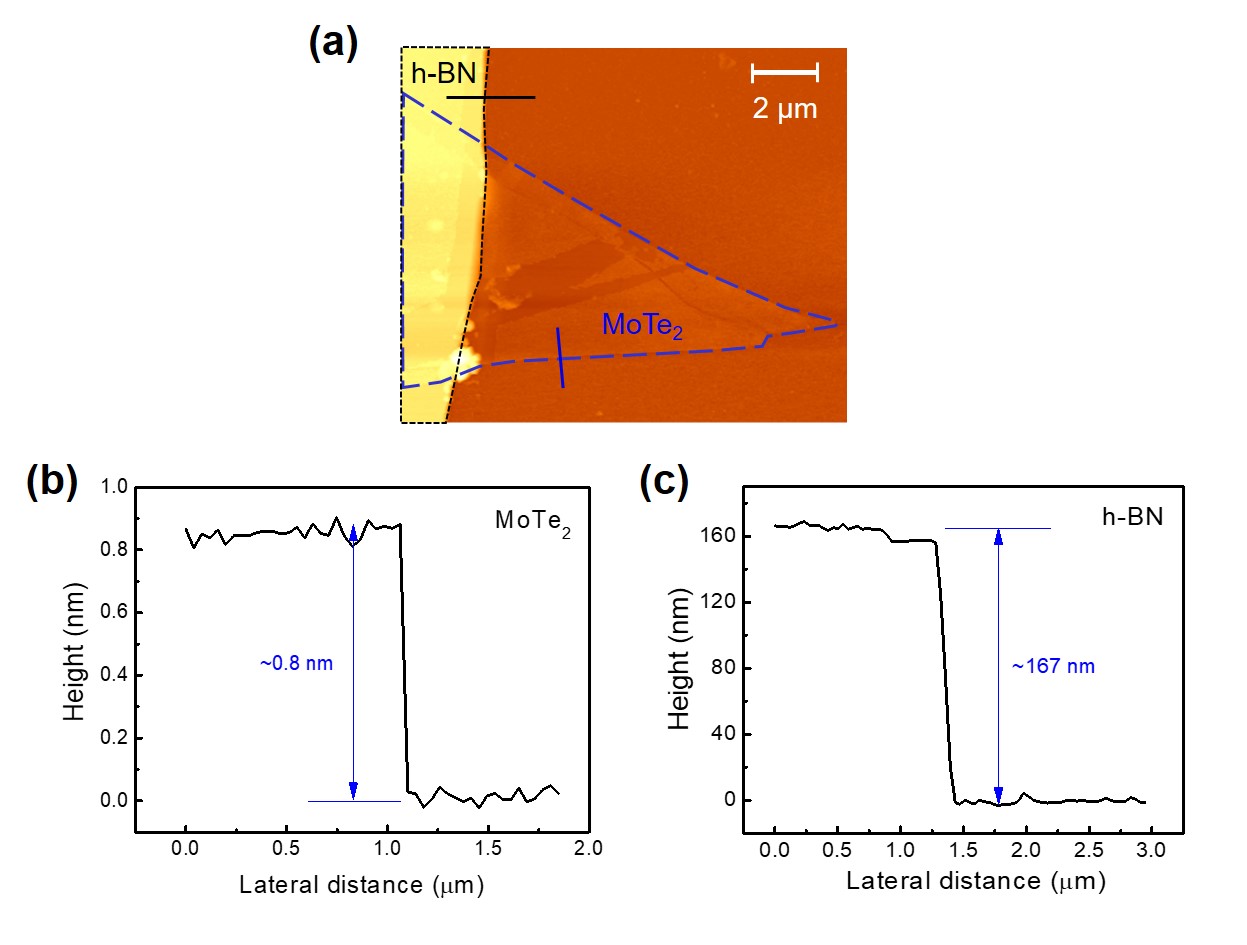
**

**Figure S2. (a)** AFM image of the MoTe_2_ FET on h-BN. **(b)** Height profile of MoTe_2_ along the blue line indicated in the AFM image (thickness ≈ 0.8 nm). **(c)** h-BN height profile, where the thickness is ~167 nm.


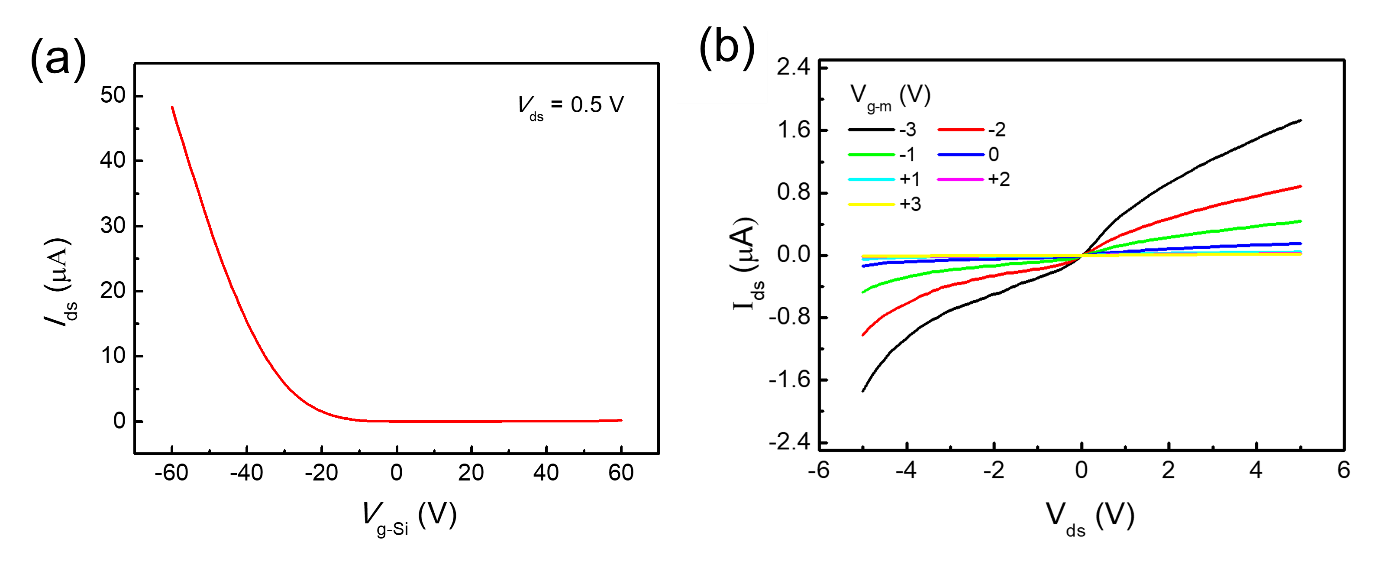


**Figure S3. (a)** The transfer curve (*I*_ds_-*V*_g-Si_) of the 0.8 nm-thick MoTe_2_ FET shows p-type behavior using Si as the back gate with sweeping from −60 to +60 V. **(b)** Output characteristics of p-type MoTe_2_ device.

**
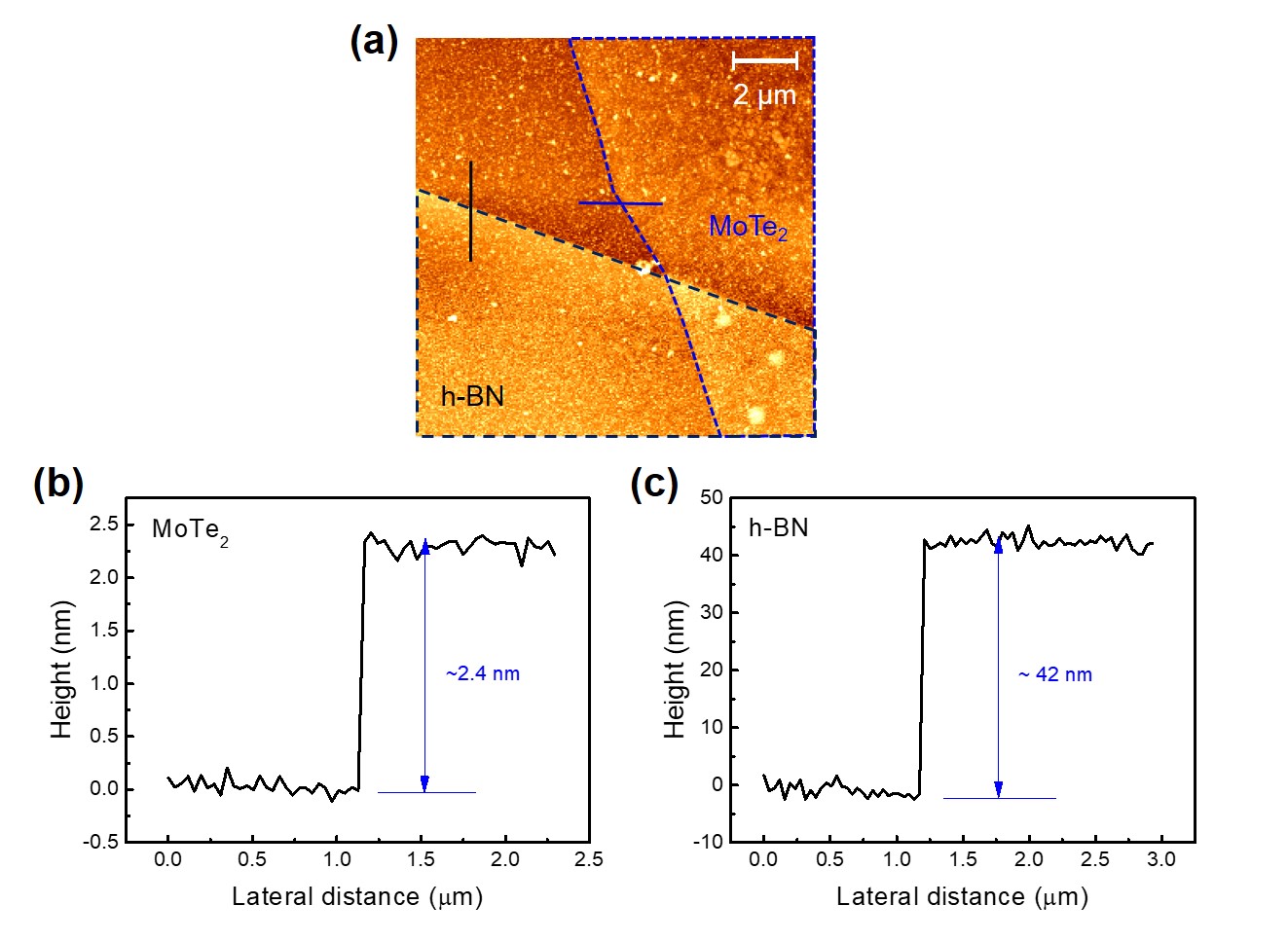
**

**Figure S4. (a)** AFM image of the MoTe_2_ FET on h-BN. **(b)** Height profile of MoTe_2_ along the blue line indicated in the AFM image (thickness ≈ 2.4 nm). **(c)** h-BN height profile, where the thickness is ~42 nm.

**
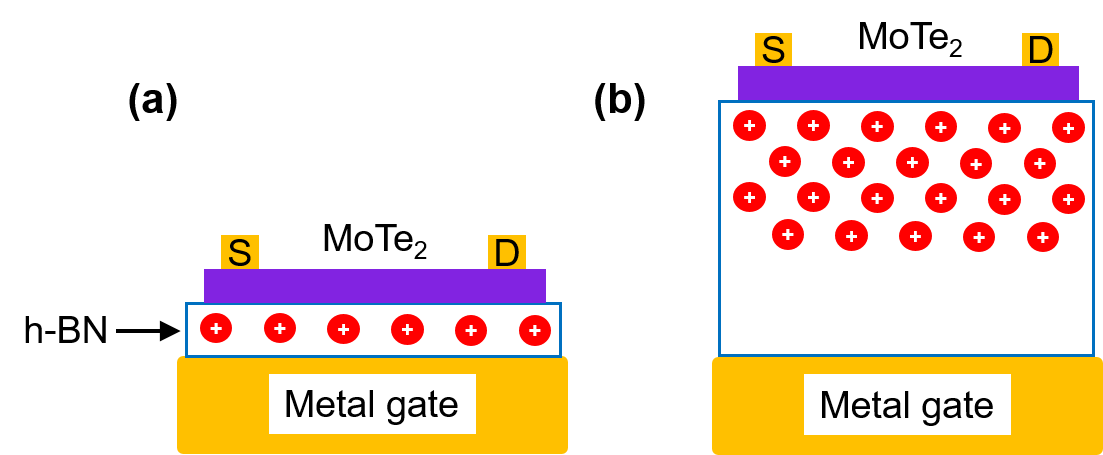
**

**Figure S5. (a)** Schematic diagram of the MoTe_2_ FET on a thin h-BN substrate after the photo-induced doping treatment, where donor-like defects are located around the upper side of the h-BN component. **(b)** Schematic diagram of the MoTe_2_ FET on a thick h-BN substrate after the photo-induced doping treatment, where the donor-like defects are distributed in the h-BN bulk.

**
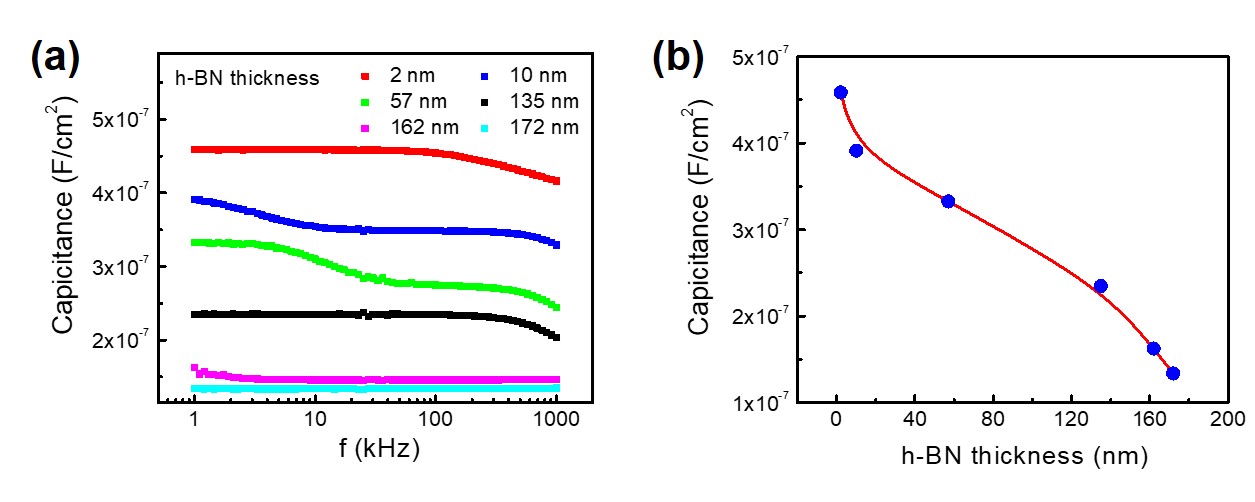
**


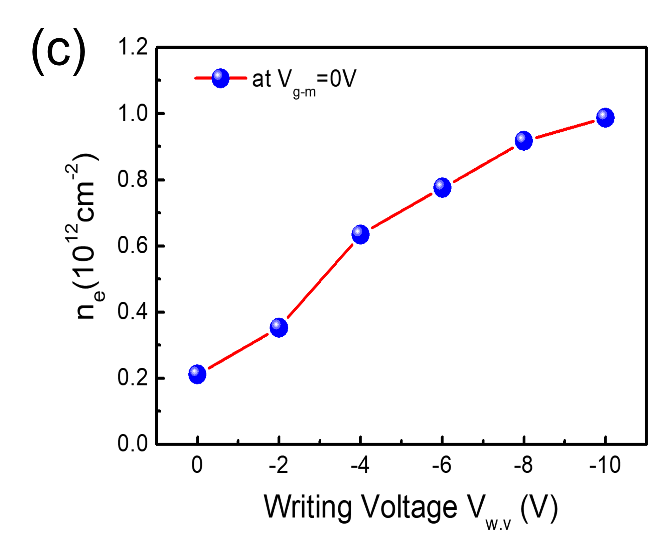


**Figure S6. (a)** Capacitance as a function of the h-BN frequency as a dielectric material. **(b)** Capacitance as a function of the h-BN thickness. **(c)** Charge-carrier concentration (n_e_) as a function of writing voltage (V_w.v_) at metal gate voltage *V*_g-m_= 0 V.


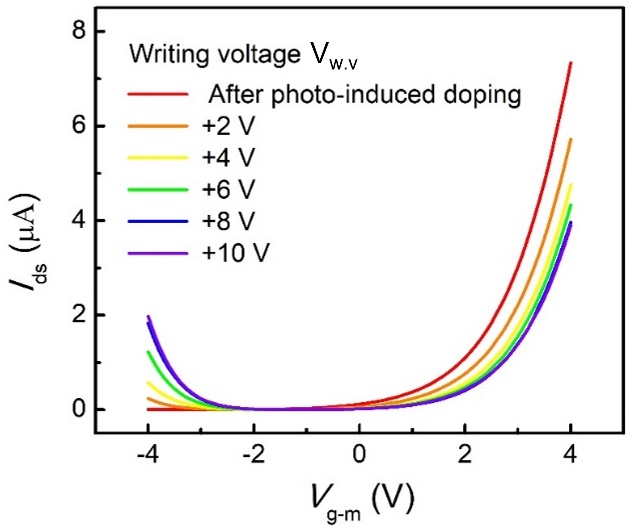


(b)


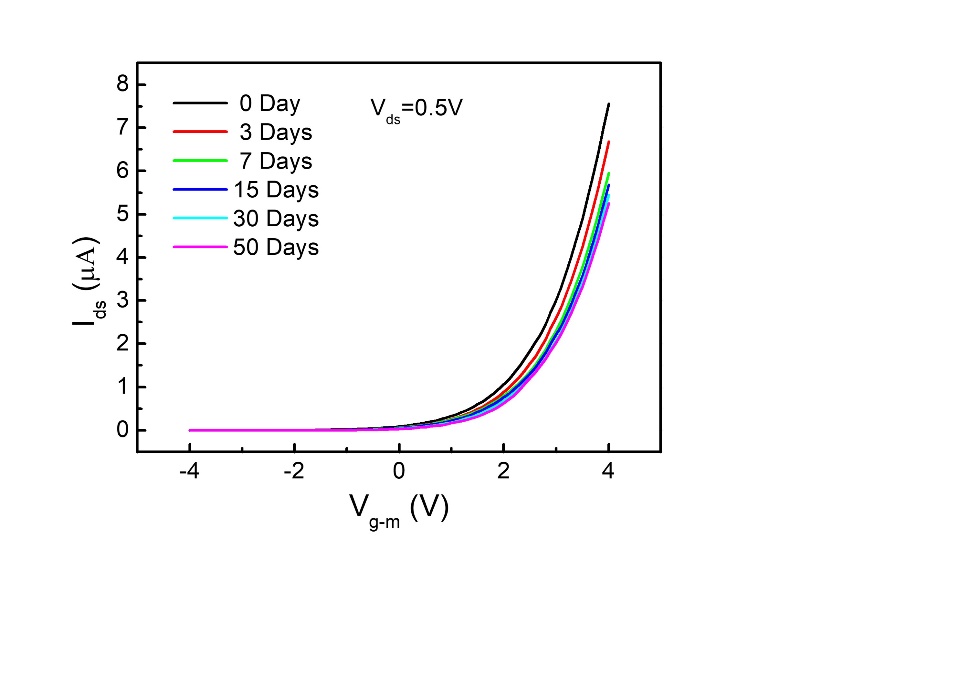


(a)

**Figure S7. (a)** The transfer characteristics of n-MoTe2 demonstrate the stability of photo-induced doping. **(b)** Transfer characteristics of the MoTe_2_ (0.8 nm) FET on a 167 nm-thick h-BN substrate after photo-induced doping for 5 min with different writing voltages. The MoTe_2_ (0.8 nm) FET on h-BN (167 nm) was prepared by photo-induced doping with the writing voltage of -10 V before starting the experiment, so that MoTe_2_ (0.8 nm) FET became n-type.


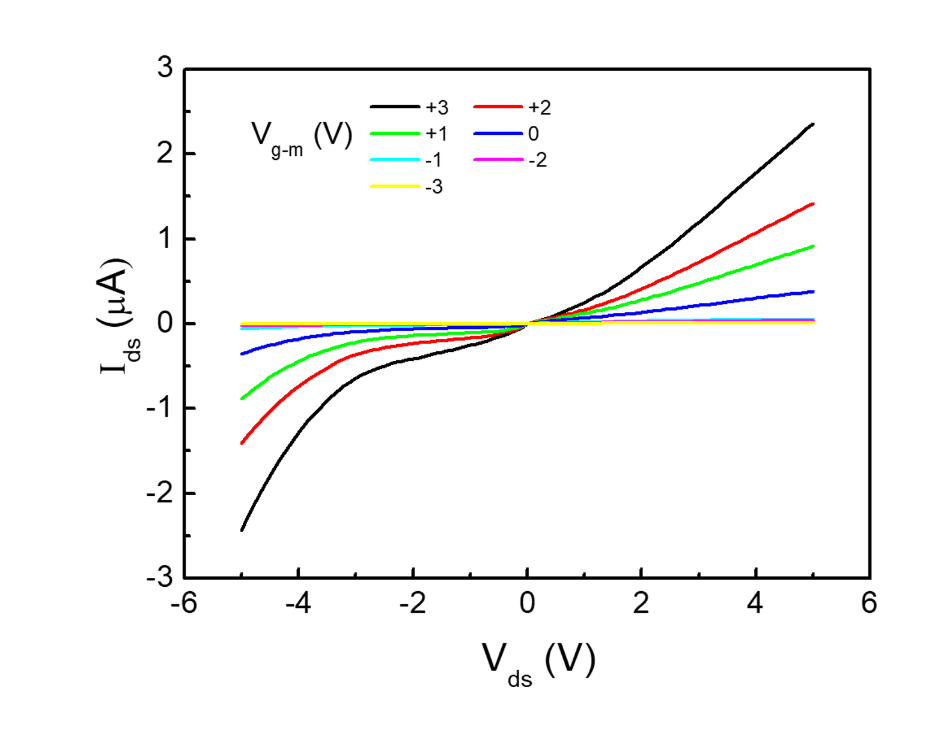


**Figure S8.** Output characteristics of pristine thick n-type MoTe_2_ device.


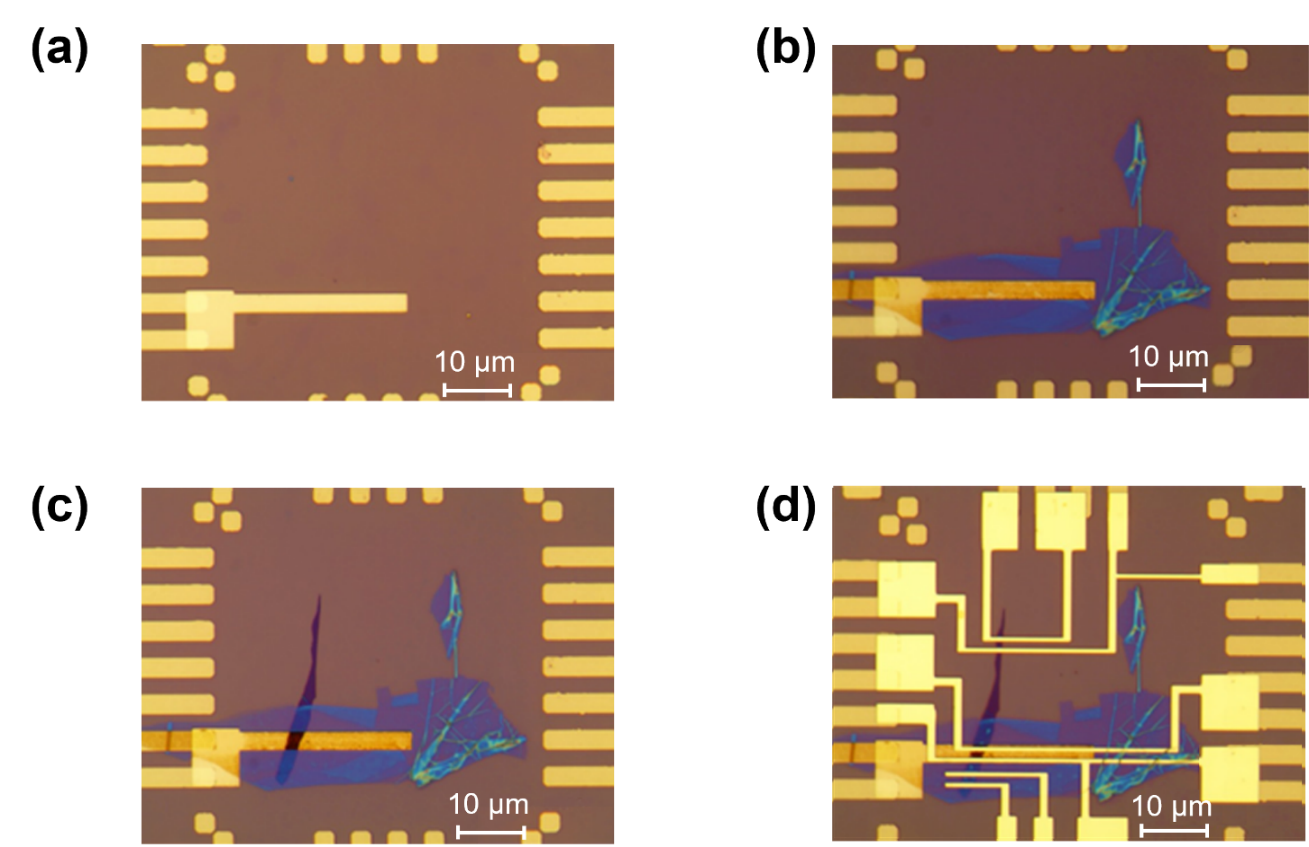


**Figure S9.** Step-by-step fabrication process of the MoTe_2_ FET on the h-BN substrate for photo-induced doping. **(a)** The lower Cr/Au (3/13 nm) electrode was fabricated for use as the writing line voltage. **(b)** The h-BN substrate was transferred onto the top of the writing voltage electrode. **(c)** A MoTe_2_ flake was transferred onto the top of the h-BN substrate. **(d)** The source and drain Cr/Au (10/80 nm) electrodes were deposited using a thermal evaporator.
